# Increased Throughput by Parallelization of Library Preparation for Massive Sequencing

**DOI:** 10.1371/journal.pone.0010029

**Published:** 2010-04-06

**Authors:** Sverker Lundin, Henrik Stranneheim, Erik Pettersson, Daniel Klevebring, Joakim Lundeberg

**Affiliations:** Division of Gene Technology, School of Biotechnology, Royal Institute of Technology (KTH), Stockholm, Sweden; George Mason University, United States of America

## Abstract

**Background:**

Massively parallel sequencing systems continue to improve on data output, while leaving labor-intensive library preparations a potential bottleneck. Efforts are currently under way to relieve the crucial and time-consuming work to prepare DNA for high-throughput sequencing.

**Methodology/Principal Findings:**

In this study, we demonstrate an automated parallel library preparation protocol using generic carboxylic acid-coated superparamagnetic beads and polyethylene glycol precipitation as a reproducible and flexible method for DNA fragment length separation. With this approach the library preparation for DNA sequencing can easily be adjusted to a desired fragment length. The automated protocol, here demonstrated using the GS FLX Titanium instrument, was compared to the standard manual library preparation, showing higher yield, throughput and great reproducibility. In addition, 12 libraries were prepared and uniquely tagged in parallel, and the distribution of sequence reads between these indexed samples could be improved using quantitative PCR-assisted pooling.

**Conclusions/Significance:**

We present a novel automated procedure that makes it possible to prepare 36 indexed libraries per person and day, which can be increased to up to 96 libraries processed simultaneously. The yield, speed and robust performance of the protocol constitute a substantial improvement to present manual methods, without the need of extensive equipment investments. The described procedure enables a considerable efficiency increase for small to midsize sequencing centers.

## Introduction

Massively parallel sequencing is currently revolutionizing sequencing data generation in biology [1]. The Genome Sequencer FLX (GS FLX) is a sequencing system generating large amounts of sequence data through massively parallel pyrosequencing [2], [3], [4]. The recent Titanium upgrade of the GS FLX sequencing system generates up to 1,200,000 reads in each run. With average read lengths of 400 bases this corresponds to outputs of up to 500 mega base pairs (bp) per run. For many applications, there is a need to be able to generate several libraries in parallel without manual intervention. Even though the sequencing capacity has been increased, the protocol for sample library preparation remains a limiting step, being laborious, expensive and time-consuming.

Sample preparation is one of the challenges associated with massive DNA sequencing [5]. Consequently, there is a need for fast, reproducible and convenient preparation methods, which are both economical and reliable. Any mistake during library preparation risks wasting precious samples, expensive reagents and time of both researchers and sequencing instruments. Automation of sample preparation has previously been shown to increase reproducibility for complex protocols [6]. Procedures to improve on library preparation for sequencing sample preparation have also been published [7], [8], [9], [10]. Precipitation of DNA in solution using polyethylene glycol (PEG) is a well-known and inexpensive method to clean up DNA, or to separate longer DNA fragments from shorter fragments, e.g. oligonucleotide primers [11], [12], [13], [14], [15]. The finding that carboxylic acid-coated superparamagnetic beads (CA-beads) could be used as solid-phase for PEG-mediated DNA precipitation made this method convenient and automatable [16].

In this study, an automated DNA library preparation method for the GS FLX Titanium sequencing system is described, which utilizes the precipitation of DNA on generic carboxylic acid-coated superparamagnetic beads as a general approach for PEG-mediated precipitation of DNA prior to sequencing. This approach can be used to readily remove shorter DNA fragments from a sample, thus cleaning it up prior to sequencing. By varying the composition of PEG in the precipitation buffer, the protocol can be adjusted to specifically suit the fragment length appropriate for the starting material, or adjusted to suit the read-length of the sequencing system. The automated protocol was evaluated by comparing it to the standard manual GS FLX Titanium library preparation protocol with respect to yield, sample throughput, robustness and sequence bias. For this task, the automated protocol was approximately two times faster, while increasing the sample throughput three-fold each run (with the possibility of running 3 preparations per day), and produced 3–5 times higher yields compared to a standard manual library preparation

## Materials and Methods

### Automation of the GS FLX Titanium Library Preparation Protocol

**Figure 1:**
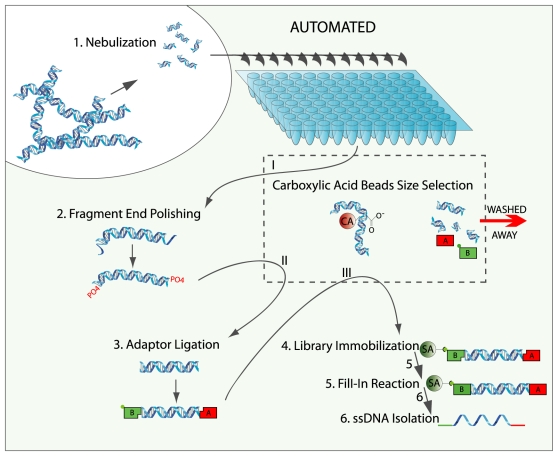
A schematic view of the automated process. Step 1–6 is the regular reaction steps of library preparation. Each sample purification is shown with roman numerals and illustrated as arrows crossing the carboxylic acid beads size selection box.

Automated library preparations were set up using a Magnatrix™ 1200 Biomagnetic Workstation (NorDiag ASA, Oslo, Norway) capable of running custom made scripts. The robot, equipped with a 12-tip head and in-tip magnet processing, is highly suitable for magnetic bead based applications and was instructed to perform all reactions as specified by the GS FLX Titanium Library Preparation Protocol. The robot also features a Peltier unit (4–95°C) where all reactions were performed. For cooling inside the instrument, but outside the Peltier unit, a PCR-cooler (Eppendorf AG, Hamburg, Germany) was used. The GS FLX Titanium Library Preparation Protocol begins with a standard fragmentation of DNA by nebulization, followed by purification and concentration with MinElute columns. The standard protocol uses AMPure® beads, calibrated to remove fragments below 400 bp, for the DNA fragment length separation after nebulization. The sample is then subjected to the following reactions and purifications in the following order: fragment end polishing, MinElute purification, adaptor ligation, MinElute purification, AMPure bead fragment length separation, library immobilization using streptavidin coated beads, fill-in reaction, and finally NaOH elution to isolate the ssDNA library containing emulsion PCR (emPCR) amplification primer sites. The automated protocol uses PEG/NaCl precipitation on MyOne™carboxilic acid-coated superparamagnetic beads (Invitrogen) as solid support for purification (CA-purification) instead of the MinElute™ (Qiagen, Hilden, Germany) purification steps and the AMPure beads for “double SPRI-method” used in the standard manual library preparation (Figure 1). Bovine serum albumin addition to end polish reaction promoted sample loss when handled by the robot due to the formation of bubbles, and was replaced by 0.1% Tween 20 (Sigma-Aldrich, St. Louis, MO, USA). In all other aspects the automated and the manual library preparations are identical.

### Evaluation of DNA Purification and Size Selection (CA-purification)

PEG 6000 (Merck, Whitehouse Station, NJ, USA) and NaCl (Merck) were dissolved in MilliQ water to a final concentration of 0.9 M NaCl and varying final PEG concentrations. The automated protocol takes 100 µl of PEG/NaCl precipitation solution to 10–100 µl MyOne™ CA-beads (washed and resuspended in 10 µl EB buffer, Qiagen), and captures the DNA in 50 µl sample. The amount of CA-beads was adjusted to the amount of DNA to precipitate, where 10 µl was seen to be sufficient for 0.5 µg of DNA (data not shown). EB buffer (Qiagen) was used to elute the samples from the CA-beads.

The fragment length necessary for precipitation was investigated by varying the final PEG concentration and precipitating DNA ladders 100–10,000 bp and 25–700 bp respectively (Fermentas, Burlington, Canada). The results were analyzed using a 2100 Bioanalyzer (Agilent Technologies, Santa Clara, CA, USA), with the DNA 7500 kit and DNA 1000 kit respectively. The yield was investigated by precipitating a 3,000 bp PCR fragment, and analyzed using a NanoDrop™ ND-1000 (Thermo Scientific, Wilmington, DE, USA).

To estimate the variance of CA-purification nebulized lambda DNA was CA-purified in 11 parallel reactions during one instrument run, and analyzed using the Bioanalyzer with the DNA 7500 kit, and concentration measurements using the NanoDrop.

### Sample Preparation

Prior to automatic and manual handling, DNA samples were nebulized using standard GS FLX library preparation nebulizers. For library preparation and sequencing, genomic DNA (from *Chironomus tentans (C. tentans)* or bacteriophage lambda) was nebulized as instructed by the manufacturer (Roche, Indianapolis, IN and 454 Life Sciences, Bradford, CT).

### Library Preparation

All library reagents were taken from GS FLX Titanium kits (Roche) except the Titanium Multiplex Identifier (MID) adaptors, which were synthesized by Thermo Scientific according to the manufacturer's specifications (Roche)(Table S1). To evaluate the robustness of yield and size distribution of the automated library preparation procedure, nebulized *C. tentans* DNA was CA-purified, pooled, and then prepared three times using the same MID adaptor for all three samples (MID3).

#### First sequencing run

To compare the automated protocol to the manual handling one sample was prepared according to Roche protocol for double-SPRI purification. The manual library was prepared using MID6 (“sample SPRI”). The automated protocol was used to prepare four samples in parallel, three samples using MID1–3 respectively (“sample 1–3”), and one using MID4 (“sample 4”). The final PEG concentration for samples 1–3 and sample 4 in the precipitation reactions were 8.1% and 7.5% respectively. The two PEG concentrations were used to evaluate the impact on sequence read length.

#### Second sequencing run

To test MID-adaptors 1–12, 12 libraries of *C. tentans* DNA were prepared in parallel, each library with a different MID-adaptor. The automated protocol included a more stringent wash routine of the immobilization beads, compared to the first sequencing library preparations to make sure that all non-immobilized DNA were removed. All preparations were analyzed with the Bioanalyzer 7500 kit prior to library preparation, and the final ssDNA libraries were analyzed with the Bioanalyzer RNA 6000 Pico kit. Sample concentrations in the final ssDNA libraries were measured using Qubit-IT ssDNA kit (Invitrogen).

### Quantitative PCR-assisted pooling

Quantitative PCR (qPCR) can be used to estimate the number of amplifiable molecules of an ssDNA library [17]. The unequal MID distribution seen in the first sequencing run led us to evaluate the relative number of amplifiable molecules between prepared libraries. QPCR was performed using an iCycler system (Bio-Rad Laboratories, Hercules, CA, USA) and iQ SYBR Green Supermix (Bio-Rad). Each library was diluted to 10^7^ and 10^6^ molecules/µl according to Qubit and Bioanalyzer measurements; these dilutions were then amplified in triplicates using 200 nM emPCR primers (forward primer 5′-CCATCTCATCCCTGCGTGTC-3′, reverse primer 5′-CCTATCCCCTGTGTGCCTTG-3′). PCR started at 94°C for 4 min, followed by 50 cycles of 94°C for 30 s, 58°C for 30 s and 72°C for 1 min 30 s. Fluorescence was measured after each cycle. The average primer efficiency (P_eff_) was calculated based on the 10-fold molecule difference of the dilutions of the libraries and the difference of cycle threshold (Ct) the samples started to amplify. (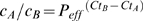, where *c_A_* and *c_B_* are the concentrations of sample A and sample B, respectively; and correspondingly Ct_A_ and Ct_B_ are the cycle times when A and B started to amplify). When P_eff_ was determined, the relative difference between the libraries was calculated from the average cycle thresholds where the 10^7^ diluted triplicates started to amplify. Nebulized *C. tentans* DNA was used as a negative control.

### Amplification and Sequencing

The emPCR titration by quantification, amplification and sequencing were performed according to the manufacturer's instructions. The manual and the automated library preparations were sequenced using a GS FLX Titanium instrument.

#### First sequencing run

Different pools of libraries were set up and sequenced on individual lanes in duplicates to compensate for loading effects. Two SV (small volume) reactions were used per sample from the SV emPCR kit (Roche). Libraries were set up as follows: a pool of all libraries (P1), a pool of sample 1–3 (P2), sample 4 individually and sample SPRI individually. A 16 lane format was used to allow assessment of each library independently, where 11 lanes where used for this study. The SPRI sample was loaded in only one lane due to poor amplification in the emPCR, resulting in only 159,000 enriched beads (Table S2) The first sequencing run generated 475,788 reads from the prepared libraries that passed all quality control filters built into the GS FLX pipeline; these reads were included in the study.

#### Second sequencing run

Two equimolar pools of all the 12 MID-libraries were set up. One pooled according to Bioanalyzer concentration measurements, and the other pooled according to the number of amplifiable molecules estimated by qPCR. The qPCR pool was sequenced on lane 1, and the Bioanalyzer pool was sequenced on lane 2. A total of two million beads were loaded on each lane resulting in 883,053 reads that passed all GS FLX quality control filters.

## Results

### DNA Purification and Size Selection

**Figure 2:**
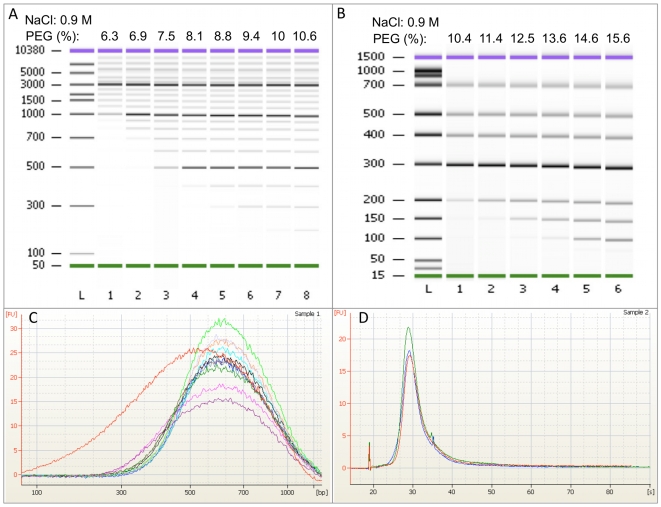
Length dependent precipitation of DNA. DNA fragment length precipitation was controlled by varying final PEG concentration (as shown at the top), while NaCl concentration was kept constant at 0.9 M. As the PEG concentration rises, smaller fragments are precipitated. A: A DNA marker ranging from 100–10,000 bp are precipitated. B: A DNA marker ranging from 25–700 bp are precipitated. C: Nebulized DNA is size-selected using CA-beads and 8.3% PEG. Samples are analyzed using Bioanalyzer DNA 7500 kit and viewed using the Bioanalyzer software, where red curve show non-size selected nebulized sample and the other colors show 11 size-selected samples. D: Three libraries are prepared in parallel from nebulized C.tentans DNA, analyzed using Bioanalyzer RNA 6000 Pico kit and viewed using the Bioanalyzer software illustrating reproducibility.

The CA-purification was performed on DNA ladders from Fermentas and nebulized bacteriophage lambda DNA to determine its robustness and the DNA precipitation length cut-off at varying final PEG concentrations. The CA-purification showed great flexibility and could handle all reaction buffers tested without need for additional purifications and without visual sample loss in the instrument. The lower limit of DNA fragment precipitation was approximately 100 bp–1,000 bp depending on the final PEG concentration (Figure 2, A and B). The yield was established to 80% when a 3,000 bp fragment was purified. The length of the DNA fragments that precipitated at a certain PEG concentration was found to be very distinct and reproducible as determined by Bioanalyzer and NanoDrop™ measurements of 11 nebulized lambda DNA samples (Figure 2C). Average concentration of the CA-purified samples as determined by NanoDrop™ was 30 ng/µl, with a standard deviation of 3 ng/µl.

### Library Preparation and Amplification

A triplicate of *C. tentans* DNA sample prepared using the automatic protocol showed very reproducible ssDNA size distributions when analyzed on the Bioanalyzer (Figure 2D). To evaluate the automated protocol further, it was compared to the standard manual GS FLX Titanium library preparation protocol using the same pool of nebulized *C. tentans* DNA. The yield of libraries produced with the automatic protocol was 3–5 times higher than manual SPRI library preparation (Table 1). The automated protocol was significantly faster than the manual procedure, processing 12 samples in parallel in 2 hours and 15 minutes with approximately 30 minutes hands-on time to prepare the robot, compared to approximately 6 hours for preparation of 4 samples using the manual procedure.

**Table 1:** Summary of the performance of the automated protocol.

	Automated	Manual
**Start**	5 µg	5 µg
**ssDNA**	150–270 ng	50 ng
**Base Quality Average**	30,4	30,4
**Read Length Average**	321 bp	322 bp
**GC content**	31%	31%

Automated library preparations are compared to SPRI manual library preparation, showing higher yield with equal average read length and base quality average from sequencing.

### Analysis of the manual and automated library preparations by sequencing

**Figure 3:**
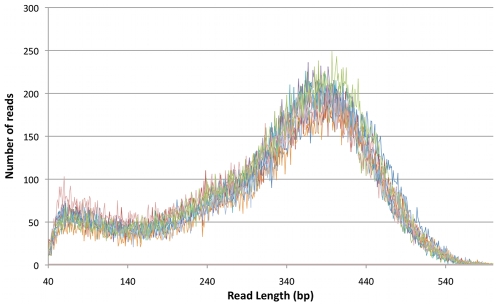
Read length distribution of the different library preparations. Fragment length is plotted against occurrence of that length. All preparations show a similar pattern.

The manual and the automated library preparations were sequenced on a GS FLX Titanium instrument to analyze the number of reads generated, GC-content, read length distributions and MID composition of the different pools. Manual and automated libraries generated comparable number of reads (Table S2). A total of 155.5 Mbp was generated from the 11 lanes used. The pool of sample 1–3 averaged at 14.4 Mbp per lane, which in a 16 lane set up would equal 230 Mbp. To evaluate library quality, average read lengths (Table 1) and length distribution were analyzed and compared between manually and automatically prepared samples, both showing comparable results (Figure 3). Sample 4 prepared with slightly higher PEG concentration showed no difference in average read length indicating that PEG precipitation concentrations within 0.6% does not significantly influence sequencing results. When libraries were pooled together, MID2 was overrepresented in terms of generated sequences, resulting in almost 60–80% of the total number of reads (Figure S1). MID3 was underrepresented with only 2–3% of the total number of reads, likely related to problems during amplification since no anomalies could be seen for any of the library preparations using this MID prior to amplification. The read length distribution for each sample all showed similar patterns (Figure S2).

### Analysis of ssDNA libraries by quantitative PCR

**Figure 4:**
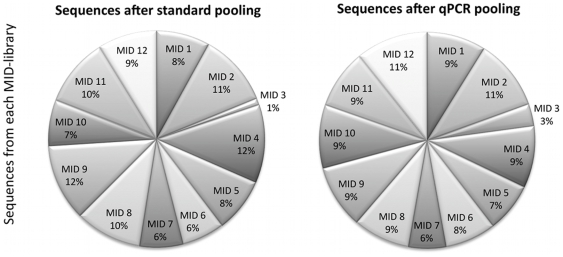
Equalizing indexed libraries. Read distribution between MID-libraries generated from the improved automatic protocol, pooled according to Bioanalyzer (left) and qPCR concentration measurements (right). The developed protocol successfully produced evenly distributed barcoded reads, except for MID3 that consistently underperformed.

QPCR was performed to analyze the uneven MID-distribution seen in the results of the first sequencing run. The concentration difference factor between amplifiable molecules in the original library dilutions of MID1, 2, 3, 4 and SPRI libraries, was determined to be 8.8, 7.4, 1.0, 6.4 and 5.6, respectively, when normalized to the least efficient library. The underrepresented MID3-tagged library after emPCR in the first sequencing run, also amplified slower during qPCR (Figure S1). For the second set of libraries prepared using 12 MIDs the amplification consistency between the libraries had been improved but MID3-tagged library still amplified slower (Figure 4). When pooling according to the qPCR results, the distribution of reads from the samples were more even, with a three-fold increase in sequences from the library tagged with MID3 (Figure 4).

## Discussion

This study describes a novel automated protocol for fast and reproducible DNA library preparation for massive sequencing using a robotic workstation capable of handing carboxylic-acid coated superparamagnetic beads for precipitation of DNA. The performance of the automated library preparation was evaluated by comparing it to a standard manual library prepared from the same source of DNA. We demonstrate that the automated library preparation for sequencing increases throughput to up to 36 samples per day, while reducing the hands-on time to 30 minutes per instrument run (Table S3). Furthermore, method training and method variation between different technicians is greatly reduced, as well as the risk for error during protocol execution. In addition, the automated library preparation outperformed manual handling up to five-fold with respect to yield of ssDNA (Table 1), most likely due to the omission of spin column purification steps. We show that our procedure is robust and to our knowledge unbiased, showing no difference in GC-content or read length when compared to the standard protocol.

The lower limit of fragment length for CA-purification can be controlled to between 100–1,000 base pairs by varying the PEG concentration in the precipitation buffer, indicating that a similar strategy can be used for purification of low molecular-weight samples.

Using an automated set up facilitates the sequencing of different samples in parallel using tagging. This allows the libraries to be sequenced simultaneously without the use of lane masking which consumes sequencing power and will fail to detect contamination or leakage between the lanes, which potentially leads to invalid interpretation of the data. With the described automated approach combined with qPCR-assisted estimation of DNA concentration, we were able to produce an even distribution of reads across barcoded samples, with the exception of one problematic barcode. Problems related to this barcode could stem from a low quality synthesis of the oligonucleotide, also supported by the constant low amplification seen in qPCR for all libraries prepared using this barcode.

Optionally, the last MinElute purification step could be removed by replacing the NaOH elution with heat elution [18]. However, this approach excludes the discrimination of fragments dually ligated with B-adaptors. This would influence subsequent concentration measurements, making this approach less suitable when pooling many samples.

An automated strategy is also feasible for other massive sequencing library preparation protocols since DNA sample concentrations steps can be replaced by the CA-purification, and most enzymatic reactions are well suited for automation in a robotic workstation. It can also assist more laborious protocols, e.g. paired end libraries, with end-polish, ligation, immobilization, fill-in-reactions etc., alleviating work load and making it possible to prepare more samples in parallel. By using two robotic workstations, an automated protocol to prepare 96 standard libraries in parallel is possible. Currently, an automated purification protocol for 96 samples using the described technique takes 1 hour and 30 minutes. In combination with similar modifications, the library preparation protocol can achieve barcoding of 96 samples in parallel. Such a level of throughput would shift the bottleneck of library preparation to sample fragmentation, since nebulization is limited to manual preparations. Another fragmentation method available is adaptive focused acoustics (Covaris), capable of automating fragmentation in a 96 well format. These methods in combination would even further alleviate the increasing need for multiplexing, as sequencing platform capacity continues to increase (e.g. for gene expression studies with multiple patients). Specifically, recent efforts by Roche to improve the library preparation protocol for GS FLX Titanium sequencing by reducing the number of steps and reagents, in combination with expected future upgrades of the instrument, likely will amount to a need for further multiplexing. The simple, flexible and robust automated protocol described in this study could easily be adapted to address this need.

In conclusion, we have demonstrated an automated DNA library preparation method for the GS FLX Titanium instrument that quickly and reproducibly generates higher yields of ssDNA compared to manual execution. Furthermore, the sample throughput of the automated protocol is up to 9 times higher than the manual protocol. The fragment size selection method we describe can be used as a general approach whenever DNA size separation needs to be performed, facilitating fast and automated handling of samples. Using the same strategy, similar automated protocols stand to alleviate workload and increase throughput for other massively parallel sequencing platforms.

## Supporting Information

Table S1 MID adaptor oligonucleotide sequences used for library preparations.
(0.13 MB PDF)

Table S2 Lane set-up from first sequencing run. Omitted lanes were used by other projects.
(0.02 MB XLS)

Table S3 Work schedule showing the preparation of 36 samples in one day. Green color indicates an automated procedure.
(0.03 MB XLS)

Figure S1 Read distribution between MID-libraries generated from standard equimolar pooling of all libraries in the first sequencing trial (gray) compared to library concentration difference factors of the individual library dilutions for those samples, determined by relative qPCR (blue) normalized to the least efficient library (MID3). The blue pie chart illustrates the predicted outcome when sequencing equal pooling of these library dilutions (percentage), based on the qPCR detected relative concentration difference (numbers).
(1.02 MB TIF)

Figure S2 Read length distribution of each prepared library from first sequencing run. MID1–4 was automatically prepared, sample SPRI (MID6) was manually prepared. Number of reads (y-axis) is plotted against read length (x-axis).
(1.68 MB TIF)
