# Supplementary material for: Increased Throughput by Parallelization of Library Preparation for Massive Sequencing
Source: PLoS One. 2010 Apr 6;5(4):e10029. doi: 10.1371/journal.pone.0010029 (PMC2850305; doi:10.1371/journal.pone.0010029)
Supplement: Table S1 — MID adaptor oligonucleotide sequences used for library preparations. (0.13 MB PDF) [file pone.0010029.s001.pdf]

**MIDs Titanium**	
Each asterisk (*) indicates a phosphorothioate-modified base.	
Bold red italics indicates 4-base key sequence.	
**TI = Titanium primer**	
Underline indicates MID sequence	
**Adaptor B**	
**Primer TIB1:**	5′-5' BioTEG-C*C*T*A*TCCCCTGTGTGCCTTGGCAGTC****T*C*A*G***- 3′
**Primer TIBprime1:**	5′-***C*T*G*A****GACT*G*C*C*A- 3′
Adaptor TIMID 1	
**TIMID 1**	5′ – C*C*A*T*CTCATCCCTGCGTGTCTCCGAC***TCAG*** ACGAGT*G*C*G*T - 3′
**TIMID1 prime:**	5′–A*C*G*C*ACTCGT***CTGA***GTCG*G*A*G*A-3′
Adaptor TIMID 2	
**TIMID 2:**	5′ – C*C*A*T*CTCATCCCTGCGTGTCTCCGAC***TCAG*** ACGCTC*G*A*C*A - 3′
**TIMID 2 prime:**	5′–T*G*T*C*GAGCGA***CTGA***GTCG*G*A*G*A-3′
Adaptor TIMID 3	
**TIMID 3:**	5′ – C*C*A*T*CTCATCCCTGCGTGTCTCCGAC***TCAG*** AGACGC*A*C*T*C - 3′
**TIMID3 prime:**	5′–G*A*G*T*GCGTCT***CTGA***GTCG*G*A*G*A-3′
Adaptor TIMID 4	
**TIMID 4:**	5′ – C*C*A*T*CTCATCCCTGCGTGTCTCCGAC***TCAG*** AGCACT*G*T*A*G- 3′
**TIMID4 prime:**	5′–C*T*A*C*AGTGCT***CTGA***GTCG*G*A*G*A-3′
Adaptor TIMID 5	
**TIMID 5:**	5′ – C*C*A*T*CTCATCCCTGCGTGTCTCCGAC***TCAG*** ATCAGA*C*A*C*G- 3′
**TIMID5 prime:**	5′–C*G*T*G*TCTGAT***CTGA***GTCG*G*A*G*A-3′
Adaptor TIMID 6	
**TIMID 6:**	5′ – C*C*A*T*CTCATCCCTGCGTGTCTCCGAC***TCAG*** ATATCG*C*G*A*G- 3′
**TIMID6 prime:**	5′–C*T*C*G*CGATAT***CTGA***GTCG*G*A*G*A-3′
Adaptor TIMID 7	
**TIMID 7:**	5′ – C*C*A*T*CTCATCCCTGCGTGTCTCCGAC***TCAG*** CGTGTC*T*C*T*A- 3′
**TIMID7 prime:**	5′–T*A*G*A*GACACG***CTGA***GTCG*G*A*G*A-3′
Adaptor TIMID 8	
**TIMID 8:**	5′ – C*C*A*T*CTCATCCCTGCGTGTCTCCGAC***TCAG*** CTCGCG*T*G*T*C- 3′
**TIMID8 prime:**	5′–G*A*C*A*CGCGAG***CTGA***GTCG*G*A*G*A-3′
Adaptor TIMID 9	
**TIMID 9:**	5′ – C*C*A*T*CTCATCCCTGCGTGTCTCCGAC***TCAG*** TAGTAT*C*A*G*C- 3′
**TIMID9 prime:**	5′–G*C*T*G*ATACTA***CTGA***GTCG*G*A*G*A-3′
Adaptor TIMID 10	
**TIMID 10:**	5′ – C*C*A*T*CTCATCCCTGCGTGTCTCCGAC***TCAG*** TCTCTA*T*G*C*G- 3′
**TIMID10 prime:**	5′–C*G*C*A*TAGAGA***CTGA***GTCG*G*A*G*A-3′
Adaptor TIMID 11	
**TIMID 11:**	5′ – C*C*A*T*CTCATCCCTGCGTGTCTCCGAC***TCAG*** TGATAC*G*T*C*T- 3′
**TIMID11 prime:**	5′–A*G*A*C*GTATCA***CTGA***GTCG*G*A*G*A-3′
Adaptor TIMID 12	
**TIMID 12:**	5′ – C*C*A*T*CTCATCCCTGCGTGTCTCCGAC***TCAG*** TACTGA*G*C*T*A 3′
**TIMID12 prime:**	5′–T*A*G*C*TCAGTA***CTGA***GTCG*G*A*G*A-3′
