# Supplementary figures and images for: Increased Throughput by Parallelization of Library Preparation for Massive Sequencing
Source: PLoS One. 2010 Apr 6;5(4):e10029. doi: 10.1371/journal.pone.0010029 (PMC2850305; doi:10.1371/journal.pone.0010029)

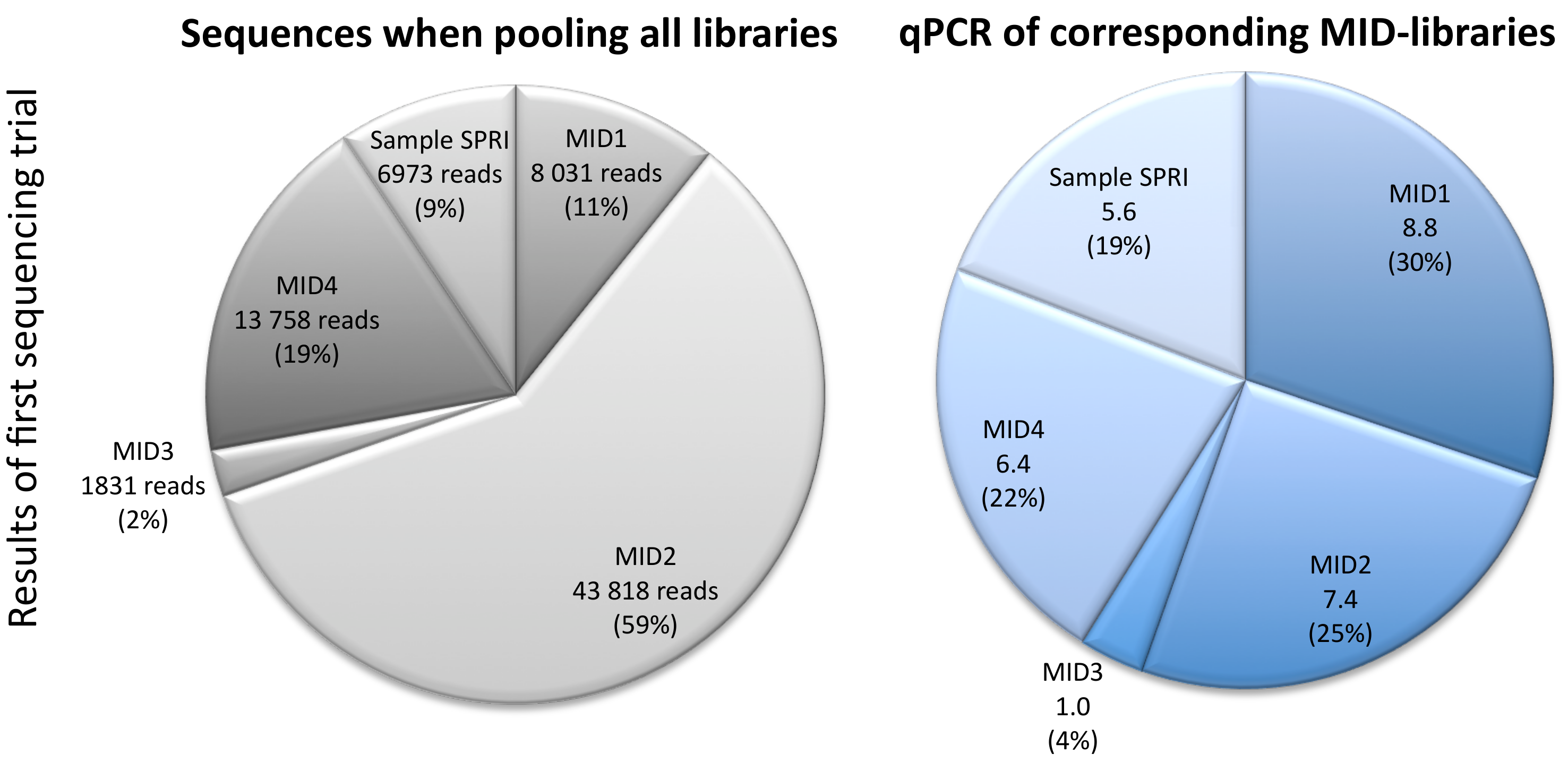

Supplement: Figure S1 — Read distribution between MID-libraries generated from standard equimolar pooling of all libraries in the first sequencing trial (gray) compared to library concentration difference factors of the individual library dilutions for those samples, determined by relative qPCR (blue) normalized to the least efficient library (MID3). The blue pie chart illustrates the predicted outcome when sequencing equal pooling of these library dilutions (percentage), based on the qPCR detected relative concentration difference (numbers). (1.02 MB TIF) [file pone.0010029.s004.tif]

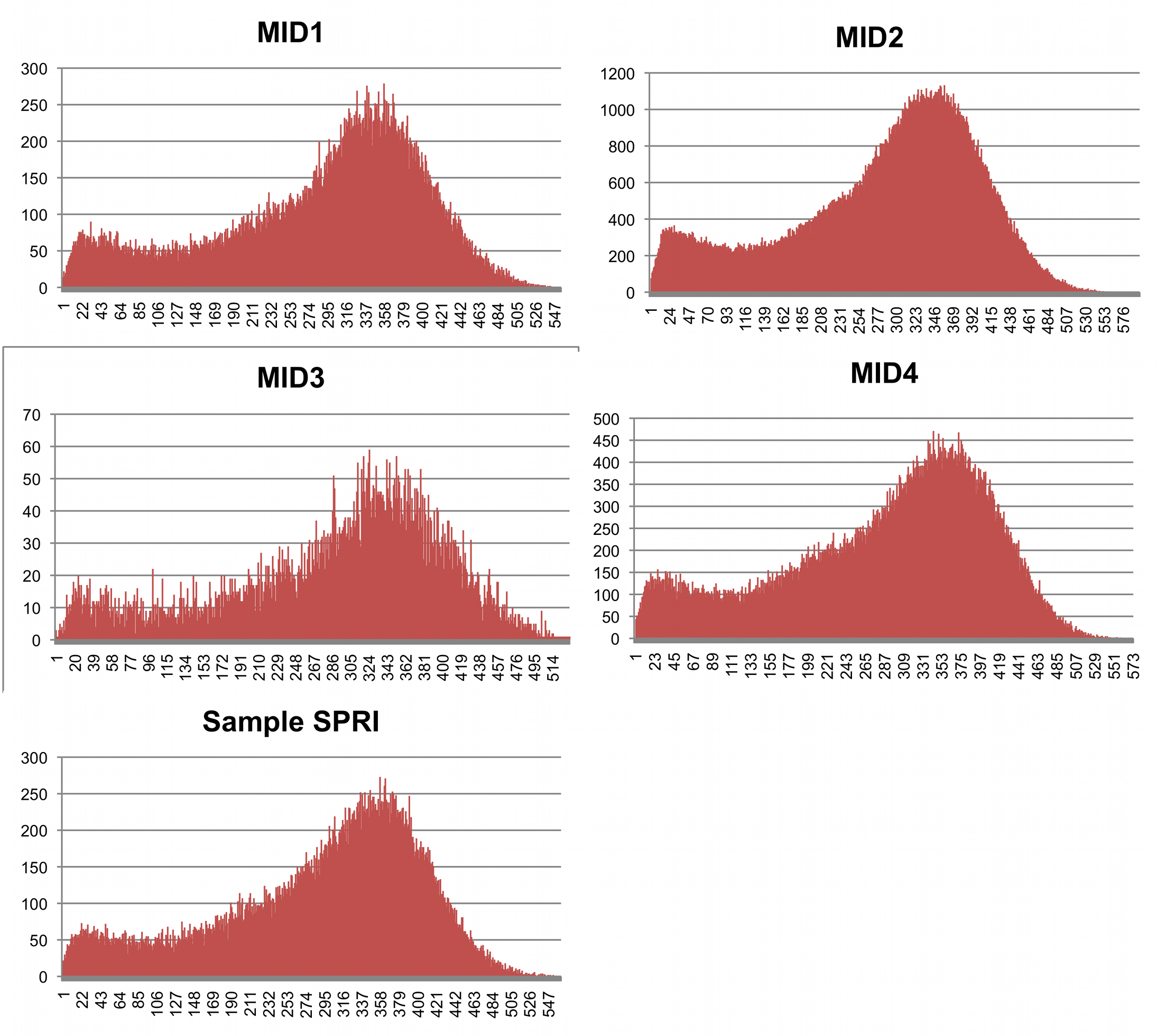

Supplement: Figure S2 — Read length distribution of each prepared library from first sequencing run. MID1–4 was automatically prepared, sample SPRI (MID6) was manually prepared. Number of reads (y-axis) is plotted against read length (x-axis). (1.68 MB TIF) [file pone.0010029.s005.tif]
